# Human Thrombin Detection Through a Sandwich Aptamer Microarray: Interaction Analysis in Solution and in Solid Phase

**DOI:** 10.3390/s111009426

**Published:** 2011-10-03

**Authors:** Alice Sosic, Anna Meneghello, Erica Cretaio, Barbara Gatto

**Affiliations:** 1 Dipartimento di Scienze Farmaceutiche, University of Padova, via F. Marzolo 5, I-35131 Padova, Italy; E-Mail: alice.sosic@studenti.unipd.it; 2 Associazione CIVEN, via delle industrie 9, I-30175 Venezia-Marghera, Italy; E-Mails: meneghello@civen.org (A.M.); cretaio@civen.org (E.C.)

**Keywords:** aptamer, thrombin, sandwich aptamer microarray

## Abstract

We have developed an aptamer-based microarray for human thrombin detection exploiting two non-overlapping DNA thrombin aptamers recognizing different exosites of the target protein. The 15-mer aptamer (TBA1) binds the fibrinogen-binding site, whereas the 29-mer aptamer (TBA2) binds the heparin binding domain. Extensive analysis on the complex formation between human thrombin and modified aptamers was performed by Electrophoresis Mobility Shift Assay (EMSA), in order to verify in solution whether the chemical modifications introduced would affect aptamers/protein recognition. The validated system was then applied to the aptamer microarray, using the solid phase system devised by the solution studies. Finally, the best procedure for Sandwich Aptamer Microarray (SAM) and the specificity of the sandwich formation for the developed aptasensor for human thrombin were optimized.

## 1. Introduction

Aptamers are RNA or single stranded DNA molecules that selectively bind to various molecular targets, such as small molecules, proteins, nucleic acids, cells, or microorganisms [1,2]. They bind to molecular targets with high specificity and affinity, making them attractive alternatives to the commonly used antibodies. Aptamers are selected *in vitro* from a large oligonucleotides pool through a process called Systematic Evolution of Ligands by EXponential enrichment (SELEX) [3]. Besides lower production costs, added advantages over antibodies are their relative ease of isolation and modification, tailored binding affinity and reversible denaturation, making them suitable candidates for use as detection systems. When adapting aptamers to a defined solid phase and to a specific detection technique, appropriate and sometimes profound post-SELEX chemical modifications must be introduced. In the case of microarrays, immobilization to the solid support and labeling for detection imply precise chemistries, depending on the physical support and detection technique, that could sensibly alter the aptamer structure [4]. In fact, since aptamers are evolved in solution, any modification altering the chemical identity of aptamer could affect its folding and consequently its binding to the target: therefore, great care must be taken when developing aptamer-based detection methods in solid phase employing aptamers previously selected in solution.

Thrombin, a protein involved in the blood coagulation cascade, was the first biological macromolecule exploited for aptamer selection [5]. Thrombin is a serine protease that plays an important role in thrombosis and hemostasis. It converts fibrinogen into clottable fibrin [6,7]. The concentration of thrombin in blood varies considerably, and can be almost absent in the blood of healthy subjects. However, it can reach low-micromolar concentrations during the coagulation process, and even low levels of thrombin can be generated in the early hemostatic process [8]. Consequently, aptamer-based assays for thrombin detection are possible diagnostic tools for monitoring the thrombin level in plasma or blood in the clinical area.

Thrombin binding DNA-aptamers have been extensively investigated [9]. In particular, the thrombin binding aptamer 1 (TBA1) and 2 (TBA2) consist of two G-quartet conformations that selectively bind to specific and different epitopes of human α-thrombin [10]. TBA1 is a 15-mer DNA aptamer which binds exosite I of thrombin (Fibrinogen Binding Site) with nanomolar affinity [11,12], while TBA2 is a 29-mer DNA aptamer binding to exosite II of thrombin (Heparin Binding Domain) with subnanomolar affinity [10]. This distinct recognition pattern allows their use in tandem, since a ternary complex could possibly be formed by simultaneous recognition of thrombin as demonstrated by several groups employing different formats and detection methods [13–15]. The paper by Huang *et al.*, in particular, reports a sandwich assay based on the two above mentioned aptamers for the detection of thrombin by time-resolved fluorescence [13]. In this case the sensing aptamer was conjugated via glutaraldehyde to a bifunctional lantanide complex, so that the signal by the europium-phenantroline-aptamer allowed for a very sensitive and specific detection of the protein. As underlined by these authors, care must be taken when covalently binding bulky fluorophores to aptamers, since an altered folding may result leading to a significant loss of affinity and specificity for the original target.

**Figure 1.**
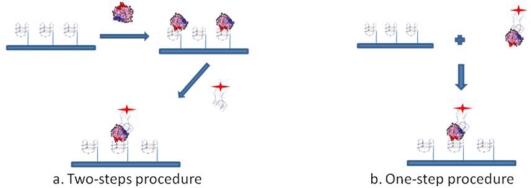
Representation of the Sandwich Aptamer Microarray (SAM) for thrombin detection performed following the two-steps **(a)** and the one-step **(b)** procedure. TBA1, anchored on the glass slide, is used as capture layer for thrombin. Fluorescently labelled TBA2 is used as detection layer. Modification on TBA1 was the introduction of 5′-NH_2_ group for aptamer anchorage on slide surface plus a polyT spacer for accessibility of the G-quadruplex to thrombin. Modification on TBA2 was the 5′-conjugation with the fluorescent label Cy5 for detection of the fluorescence signal by a laser scanner.

To investigate whether post-SELEX chemical modifications introduced in aptamers would affect thrombin recognition prior to the development of a Sandwich Aptamer Microarray (SAM) system, we adopted these two aptamers and human thrombin as a model system, with the aim of recognizing in solution by a simple gel electrophoresis method any perturbation in the complex formation before the solid phase development. Following the indications of the solution studies, we adopted the SAM system, depicted in Figure 1, making use of laser scanning for the fluorescent detection of the thrombin-aptamer sandwich on the microarray.

The chemical modifications introduced in the capture aptamer TBA1 were: a 5′-NH_2_ group, needed for aptamer anchorage on slide surface and a polyT spacer also introduced at the 5′-end for making the G-4 motif of the anchored aptamer more accessible to the target protein, as indicated by the work of Lao *et al.* [16]. In the case of the secondary (sensing) aptamer (TBA2) a fluorescent dye, namely Cy5, was directly conjugated to the 5′-end of the oligonucleotide.

The analysis in solution on the complexes formation between human thrombin and the modified aptamers was performed on native polyacrylamide gels by Electrophoretic Mobility Shift Assay (EMSA), also referred as gel shift assay, and the verification of the sandwich complex formation in solution by a Supershift Assay, incubating simultaneously the two aptamers with the target protein. The validated system was finally applied to the solid phase using an appropriate control and two different protocols for detection. The results obtained in the microarray are positive and consistent with the results obtained by the analysis in solution, and constitute the proof of principle of our approach of validating in solution the effects of chemical modification of aptamers prior or along the development of the solid phase.

## 2. Experimental Section

### 2.1. Aptamer and Thrombin Solutions

The sequence of the unmodified 15-mer TBA1 [11] was: 5′-GGT TGG TGT GGT TGG-3′. TBA1 was used in its unmodified form as control; to allow immobilization on solid phase, a 5′-amino modification (TBA1-NH_2_) and a 5′-amino modification plus a polyT(12) tail as spacer [reported as TBA1(12T)NH_2_] were also synthesized. The sequence of the unmodified 29-mer TBA2 control (TBA2) [10] was: 5′-AGT CCG TGG TAG GGC AGG TTG GGG TGA CT-3′. TBA2 was used as 5′-Cy5 labeled (TBA2Cy5) to allow detection. As negative control in the Sandwich Aptamer Microarray we used an aptamer (OTA) that binds with high affinity and specificity to ochratoxin A [17]; the sequence of 5′-amino modified OTA to allow the binding on the slide was: 5′-NH_2_-GAT CGG GTG TGG GTG GCG TAA AGG GAG CAT CGG ACA-3′. All the aptamers were purchased from IDT Integrated DNA Technologies (Munich, Germany) and stored at −20 °C in TE (10mM Tris-HCl, 1mM EDTA) pH 8.0. Human α-thrombin (T1063) was purchased from Sigma-Aldrich (St. Louis, MO, USA), dissolved in deionized purified (MilliQ) water, aliquoted and stored as recommended by the supplier.

### 2.2. EMSA (Electrophoresis Mobility Shift Assay) Analysis for Binary Complexes

The binding of each aptamer to the protein target in solution was analyzed by EMSA. Prior to incubation with thrombin, all the aptamer samples (oligonucleotide 10 μM in presence of KCl 100 mM) were denatured at 95 °C for 5 minutes and the samples were left to cool to room temperature (slow annealing). Each folded aptamer (1 μM) was then incubated with increasing concentrations (from 0 to 12 μM) of human thrombin in a total volume of 20 μL, at 25 °C for 30 min. After incubation, free aptamer and thrombin-aptamer complexes were resolved by 12% non-denaturing polyacrylamide gels containing 1X TBE buffer (Tris-HCl 89 mM, borate 89 mM, EDTA 2mM) and KCl 10 mM. Aptamers on the gels were stained with the fluorescent DNA binding dye SybrGreen II^®^ (Invitrogen) that preferentially binds to single-stranded DNA and emits fluorescence (λ_exc_ = 488 nm; λ_em_ = 522 nm) when in complex with DNA. Fluorescence in gel systems was detected on a Geliance 600 Imaging System (PerkinElmer).

### 2.3. Supershift Assays for Ternary Complexes

To verify the sandwich formation in solution a Supershift Assay was performed: both pairs of folded aptamers (each 1 μM) were incubated simultaneously with the protein (5 μM) in the Binding Buffer (Tris 20 mM, KCl 5 mM, NaCl 140 mM, MgCl_2_ 1 mM, pH 7.5) in a total volume of 20 μL, at 25 °C for 30 min. After incubation, free aptamers and thrombin-aptamers complexes were resolved by 12% non-denaturing polyacrylamide gels containing 1X TBE buffer (Tris-HCl 89 mM, borate 89 mM, EDTA 2mM) and KCl 10 mM. Aptamers on the gels were stained also in this case with the fluorescent DNA binding dye SybrGreen II^®^ (Invitrogen) that preferentially binds to single-stranded DNA and emits fluorescence (λ_exc_ = 488 nm; λ_em_ = 522 nm) when in complex with DNA. Fluorescence in gel systems was detected on a Geliance 600 Imaging System (Perkin Elmer). Relative mobilities of each band were calculated as Rf, *i.e.*, the ratio between the distance traveled by the sample (band) divided by the distance traveled by the marking dye (bromophenol blue) in the gel.

### 2.4. Aptamer Preparation for SAM

In the aptamer arrays TBA1 was used as capture layer for thrombin. A sequence that binds with high affinity and specificity to ochratoxin A, was anchored as negative control on a distinct spot on the slide surface parallel to TBA1’s spots. Prior to immobilization on the glass slide, TBA1 and OTA (80 μM in presence of KCl 100 mM) were denatured at 95 °C for 5 minutes and then left to cool to room temperature. This folding step ensures that they assume the G-quadruplex structure, responsible for thrombin binding. Folded aptamers were then diluted in the Printing Buffer 1.5X (Printing Buffer 6X: sodium phosphate300 mM, 0.02% Triton pH 8.5) to a final concentration of 20 μM. These probes were loaded into micro-plates and submitted to the Spotter Arrayer (Versarray Chip Writer Pro System, BioRad) for slide printing.

### 2.5. Printing of Aptamer Microarrays

E-surf LifeLine slides (25 mm × 75 mm, LifeLineLab, Pomezia, Italy) were used for printing: these slides are obtained by adsorption on glass of a hydrophilic polymer containing N,N-acryloyloxysuccinimide (NAS), allowing binding to the 5′-amino modified DNA aptamers TBA1-NH_2_ and OTA. To improve capture-protein efficiency, we anchored on the glass slide also the TBA1 5′-amino modified with a polyT (12T) spacer at the 5′-end. In this way the G-quadruplex structure of TBA1 can fold correctly to recognize the protein target.

The same spot scheme shown in Table 1 was printed 12 times (12 sub-arrays) on the same slide: capture aptamers [TBA1-NH_2_ or TBA1(12T)NH_2_] and OTA control were printed in six spots to ensure the reproducibility of the system. Slides were printed by the Spotter Arrayer instrument, using Telechem SMP3 microspotting pins. Printed slides were incubated overnight in a 75% humidity incubation chamber, blocked at 30 °C in Blocking Solution (0.1 M Tris, 50 mM ethanolamine), washed at 30 °C in Washing Solution (4X SSC, 0.1% SDS) and finally washed in MilliQ H_2_O, spin-dried and stored properly until usage. Each slide has the possibility to test up to 12 samples at once, since each sub-array can be physically isolated from the others by the Corning Microarray Hybridization Chamber (Corning, NY, USA) during the hybridization phase of the experiment. This approach ensures to hybridize up to 12 samples at the same time and nevertheless to minimize array variation resulting from minor fluctuation of external parameters.

### 2.6. Protein Labeling

The AlexaFluor^®^555 monoreactive Succinimidyl Esters (Invitrogen), dissolved in anhydrous dimethyl sulfoxide (DMSO) solvent was used to label thrombin samples. Prior to labeling, thrombin and AlexaFluor^®^ 555 were first diluted to proper reaction concentrations in MilliQ H_2_O. The labeling reaction was done mixing thrombin samples with the AlexaFluor^®^555 NHS esters reagent in the Coupling Buffer (sodium carbonate 0.1 M, pH 9.4); the optimal dye to protein ratio was 2; the reaction was incubated 2 h at 4 °C. After this time, glycine was added to the mixture in order to block the free unreacted AlexaFluor^®^555 NHS esters. The labeled protein samples were kept in the dark at 4 °C, and all samples were used within a week.

### 2.7. Detection Layer Preparation

The fluorescently labeled TBA2 (TBA2Cy5) was used as secondary aptamer for detecting thrombin captured by the primary aptamer (TBA1). TBA2Cy5 (10 μM in KCl 100 mM) was denatured at 95 °C for 5 minutes and then left to cool to room temperature in order to assume G-quadruplex structure, which is essential for recognition of the heparin binding domain of thrombin.

### 2.8. Sandwich Aptamer Microarray Assays

The printed aptamer microarrays prepared as detailed above were immersed just before analysis in the Binding Buffer 1X (Tris 20 mM, KCl 5 mM, NaCl 140 mM, MgCl_2_ 1 mM, pH 7.5) at room temperature for 15 min.

The sandwich aptamer microarrays were performed with two procedures, *i.e.*, analyzing the sandwich formation on surface either in one step or in a two steps protocol (see Figure 1). The one step-procedure consisted in the pre-incubation of thrombin with the fluorescently labelled aptamer (TBA2Cy5) in solution in the Binding Buffer, at 25 °C for 30 min. The pre-formed complex thus obtained was then incubated in the microarray at 25 °C for 30 min. The two steps-procedure consists of the incubation in the microarray of thrombin diluted in the Binding Buffer at 25 °C for 30 min, followed by the incubation with the fluorescently labelled secondary aptamer (TBA2Cy5) diluted in the Binding Buffer, at 25 °C for further 30 min. Finally, the aptamer microarrays were carefully rinsed with the PBS Buffer (phosphate 10 mM, NaCl 137 mM, KCl 3 mM, pH 7.4), washed three times with PBS at room temperature to remove the unbound proteins and rapidly rinsed in MilliQ H_2_O before spin-drying, ready to be scanned.

### 2.9. Slides Scanning and Data Analysis

Spin-dried aptamer arrays were scanned using Genepix 4000B laser scanner (Molecular Devices) and the Gene Pix Pro software using both 532 nm and 635 nm wavelength and PTM gain 550 for both channels (Power 33). Fluorescent spot intensities were quantified using the Gene Pix Pro software after normalizing the data by subtracting local background from the recorded spot intensities. For each set of six spots median and standard deviation were calculated.

## 3. Results and Discussion

### 3.1. Analysis and Optimization of TBA1-Thrombin Interaction

#### 3.1.1. Role of K^+^ Ions

**Figure 2.**
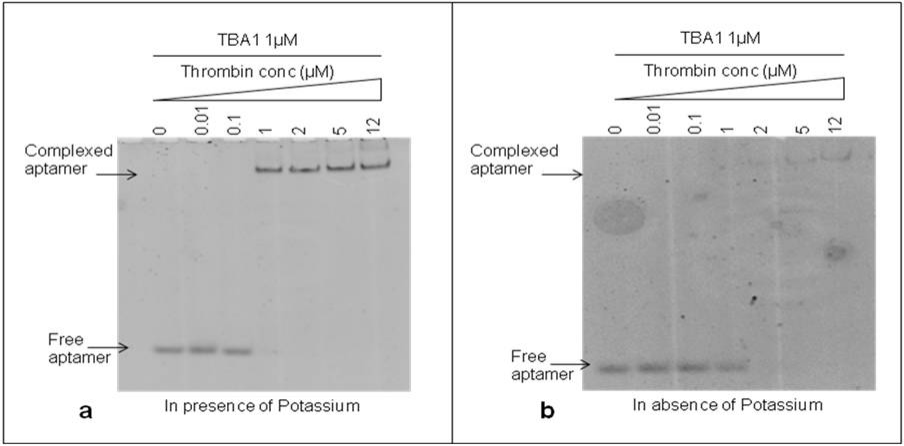
Electrophoretic Mobility Shift Assay (EMSA) of unmodified TBA1 and thrombin in presence **(a)** and in absence of Potassium **(b)**. TBA1 was incubated with the protein under the conditions described. We have indicated in the figure the respective TBA1 and thrombin concentrations. A control reaction without thrombin was performed in all experiments. KCl was added to the polyacrilamide gel and to the running buffer. Binding reactions were applied on a 12% non-denaturing PAA gel containing 1X TBE buffer and KCl 10 mM. The mobility of free and complexed aptamers, stained by SybrGreen II®, was detected using the Geliance 600 Imaging System.

The DNA 15-mer TBA1 recognizes specifically the Fibrinogen Binding Domain (FBD) of thrombin by adopting the four-stranded DNA structure known as G-quadruplex (G-4) [11,18]. Monovalent cations, in particular K^+^ and Na^+^, are essential to form and stabilize G-4 structures [19], with K^+^ ions favoring the recognition of thrombin to immobilized aptamers at temperatures higher than 25 °C [20]. For TBA1 and TBA2 folding of TBA1 into G-quartets in solution is favored by the presence of K^+^ ions, being 5.8 mM the minimal concentrations required for TBA1, while TBA2 requires a slightly higher ion concentration [21]. Therefore, we investigated the effect of potassium ions 10 mM on the binding of TBA1 and thrombin in our conditions to decide whether it would be a component of the buffer system. TBA1 was denatured, folded and incubated with thrombin (up to 12 μM) in the presence or absence of KCl. Complex formation was analyzed by EMSA. Free and complexed aptamers were detected in the gel by staining with SybrGreen II. As shown in Figure 2(a), the band corresponding to free TBA1 shifts up in the gel starting from 1:1 TBA1/thrombin ratio, consistent with the lower mobility of the TBA1-thrombin complex. At the same time, the free TBA1 band disappears. The presence of the upper band with lower electrophoretical mobility indicates that TBA1 is complexed with the target protein under these conditions, *i.e.*, in the presence of K^+^ ions. Figure 2(b) shows the result of the same experiment performed in the absence of K^+^ ions at all steps (folding, incubation with thrombin and analysis by EMSA). We can observe that TBA1 binds thrombin but with reduced affinity, since the shift of TBA1 band appears at a higher TBA1/thrombin ratio (1:2). Although thrombin itself is known to act as a molecular chaperone promoting the G-quadruplex formation in the aptamer [22], the presence of K^+^ cations favours G-4 formation and facilitates TBA1-thrombin binding. Higher concentrations of potassium ions were also analyzed (up to 100 mM), but the high salt in the gel and in the running buffer impairs EMSA assay. For these reasons we considered as optimal binding conditions the presence of K^+^ ions and 10 mM KCl was included in our systems.

#### 3.1.2. Role of TBA1 Modifications

**Figure 3.**
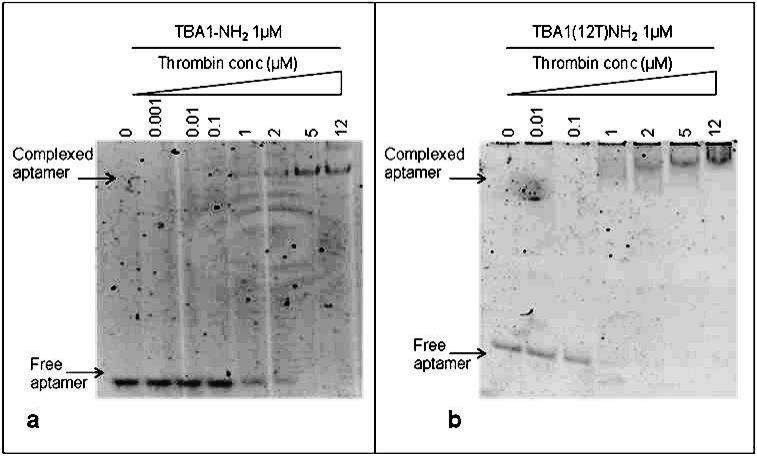
Electrophoretic Mobility Shift Assay (EMSA) of TBA1-NH_2_ with thrombin **(a)** and of TBA1(12T)NH_2_ with thrombin **(b)**. TBA1-NH_2_ and TBA(12T)NH_2_ were separately incubated with the protein under the conditions described. We have indicated in the figure the respective aptamer and thrombin concentrations. A control reaction without thrombin was performed in all experiments. Binding reactions were applied on a 12% non-denaturing PAA gel containing 1X TBE buffer and KCl 10mM. The mobility of free and complexed aptamers, stained by SybrGreen II®, was detected using the Geliance 600 Imaging System.

To immobilize TBA1, the capture layer in our aptamer-based microarray, 5′-amino modification of the oligonucleotide was required for aptamer anchorage on the glass slides. Therefore, we investigated in solution the effect of this modification on thrombin binding: folded TBA1-NH_2_ (1 μM) was incubated with increasing concentrations (0, 0.001, 0.01, 0.1, 1, 2, 5 and 12 μM) of human thrombin following the procedure described above for unmodified TBA1. Figure 3(a) shows that, starting from a 1:1 aptamer to thrombin ratio, the band of the free aptamer gradually disappears and at the same time the band with lower mobility corresponding to TBA1-NH_2_-thrombin complex appears. Hence, comparing these results with what seen for unmodified TBA1 [Figure 2(a)], the 5′-amino modification does not impair the TBA1-thrombin binding. To evaluate the effect of the polyT spacer added at the 5′-end of the oligonucleotide, the same experiment was performed using TBA1(12T)NH_2_: the results are reported in Figure 3(b).

From a 1:1 aptamer/thrombin ratio the band of the free aptamer shifts up in the gel in bands that are slightly smeared compared to those of unmodified aptamer-thrombin [compare Figure 3 with Figure 2(a)]. We can conclude that, although more than one complex seems to be present relative to the unmodified control, suggesting the possible presence of different folding equilibria, the addition of the polyT (12T) spacer at the 5′-end of the 15 nucleotides capture aptamer TBA1 does not inhibit the recognition of the protein in solution, in agreement with what reported in solid phase for the recognition of Cy-labeled thrombin with the modified aptamer [16].

### 3.2. TBA2-Thrombin Interaction in Solution

#### 3.2.1. Evaluation of Labeling with Cy5 Dye

**Figure 4.**
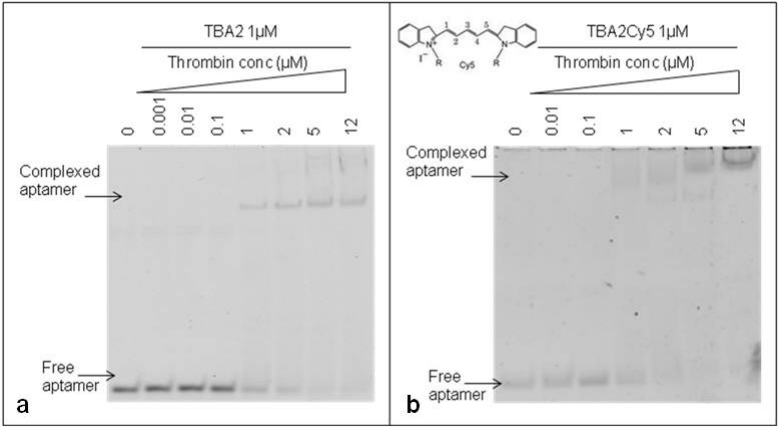
Electrophoretic Mobility Shift Assay (EMSA) of unmodified TBA2 with thrombin **(a)** and TBA2Cy5 with thrombin **(b)**. Each aptamer was incubated with the protein under the conditions described. We have indicated in the figure the respective aptamer and thrombin concentrations. A control reaction without thrombin was performed in all experiments. Binding reactions were applied on a 12% non-denaturing PAA gel containing 1X TBE buffer and KCl 10 mM. The mobility of free and complexed aptamers, stained by SybrGreen II^®^(a,b) was detected using the Geliance 600 Imaging System.

The 29-mer DNA TBA2, planned to be used for fluorescence detection in our aptamer-based microarray, has to bear important chemical modifications at its 5′ end: to rule out any possible interference of the aromatic systems of fluorescent dyes on the folding and on the recognition of the aptamer with the target, thrombin binding by the unmodified TBA2 was compared to that exhibited by TBA2 fluorescently labeled at the 5′-end with the red light emitter Cy5 (TBA2Cy5). The gel shifts of the unmodified and modified aptamers are shown in Figure 4.

The unmodified TBA2 in KCl 10 mM folds in G-quadruplex, as reported by CD titrations [21], and binds thrombin from TBA2/thrombin ratio 1:1 [Figure 4(a)]: the band corresponding to TBA2 shifts up and new bands with lower mobility, representing the TBA2-thrombin complex, appear in the upper part of the gel, as expected. An evident gel shift at same ratio aptamer/protein was obtained even when the 5′-Cy5 labeled TBA2 was analyzed [Figure 4(b)]. The 5′-labeling of TBA2 with Cy5 evidently influences the overall hydrodynamic volume of the complex with thrombin, as seen by the appearance of bands of relative lower mobilities [Figure 4(b)] compared to the complex with unlabeled aptamer controls [Figure 4(a)]. The planar and hydrophobic surfaces individuated by the fluorophore and, possibly, also the structure(s) of the binary complex(es) with thrombin are affected: at the highest protein/oligo ratio the modified aptamer forms complexes with even lower electrophoretic mobilities, possibly consistent with the formation of aptamer/protein complexes with a different stoichiometry or a different shape. These observations are in line with literature data on the strict structural requirements of TBA2 for correct folding and target binding [23]. However, we can observe that the presence of the labeling dye Cy5 conjugated to the aptamer allows thrombin recognition, since the binary complex formation is not abolished. The solution experiments point to the requirement of careful handling of the detection aptamer when adapting the solution studies to the solid phase: correct structuring of the labeled TBA2 must be ensured by denaturing/folding protocols prior to its use to ensure thrombin recognition.

### 3.3. Supershift Assay: Optimization of TBA1-Thr-TBA2 Interaction

After having completed the studies of thrombin binding for each set of modified aptamers, we verified the formation of the ternary complex in solution. The Supershift Assay using the aptamers with the modifications needed for solid phase will indicate the feasibility of the sandwich system in the planned microarray and will constitute the proof of principle of our approach.

The Supershift Assay was performed with TBA1(12T)NH_2_ as capture layer and TBA2Cy5 as detection layer. The primary aptamer and the secondary aptamer were folded in KCl, mixed and then incubated with thrombin(s) in the Binding Buffer at 25 °C for 30 min. Human thrombin and Alexa555-labeled human thrombin were used to verify ternary complex formation. After incubation, free aptamer and thrombin-aptamer complexes were resolved by 12% non-denaturing polyacrylamide gel containing KCl. Free aptamers and the binary complex of each aptamer with thrombin were loaded in the gel as controls.

**Figure 5.**
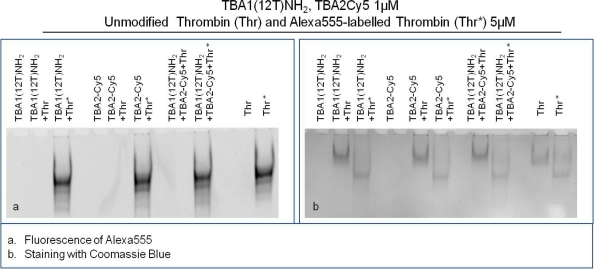
Electrophoretic Mobility Supershift Assay (EMSA) of TBA1(12T)NH_2_ + thrombin + TBA2Cy5: detection of Alexa555-labeled thrombin **(a)**; detection of thrombin with Coomassie Blue staining **(b)**. Each aptamer was incubated separately or simultaneously with the unmodified and fluorescently labeled protein under the conditions described. We have indicated in the figure the respective aptamer and thrombin concentrations. A reaction control without thrombin was performed for each aptamer; other reaction controls were the unmodified and fluorescently labelled thrombin incubated without any aptamer. Binding reactions were applied on a 12% non-denaturing PAA gel containing 1X TBE buffer and KCl 10 mM. The mobility of free and complexed aptamers was detected using the Geliance 600 Imaging System.

The results of this experiment employing different detection systems are reported in Figure 5. Figure 5(a) shows the image detected analyzing green light emission without any gel staining: the bands detected correspond to thrombin labelled with Alexa555. Figure 5(b) shows the result of the supershift assay using Coomassie Blue to visualize the position of unlabeled thrombin and Alexa555 labeled thrombin, either alone or complexed with aptamers. A third method of staining with the fluorescent intercalator SybrGreen II (not shown), allows the detection of the aptamer bands in the gel.

Thrombin has an almost neutral pI [20] and its mobility is limited in the native gel system employed (Tris borate buffer, pH 8.3). It is clearly evident in Figure 5(b) that Alexa labeling changes the mobility of the protein: Alexa-thrombin moves faster compared to unmodified thrombin. Although the molecular structure of Alexa555^®^ is not disclosed by the manufacturer, it possesses a negative charge (information provided by Invitrogen) that alters the charge profile of the protein, and is responsible for the higher mobility of the protein in the native polyacrylamide gel system used.

The band of each free aptamer shifts up in presence of fluorescently labeled or unmodified thrombin, due to the higher molecular weight aptamer-protein complex formation, as verified in the previous experiments. When in complex with both aptamers thrombin forms a ternary complex verified by the appearance of the supershift band with lower mobility than the binary complex band [Figure 5(b)]. This is clearly evident in the case of unmodified thrombin, and it is also verified for Alexa-thrombin. The relative mobilities of the complexes, calculated as Rf for each band, are summarized in Table 2.

Alexa-labeled protein moves faster than the unlabeled thrombin (Rf: 0.09 *vs.* 0.07); in the case of binary complexes with human thrombin the bands are shifted up from the protein alone (to 0.05 for both capture and detection aptamer), and are further shifted (to 0.04) when all the system players are in a ternary complex. With Alexa-thrombin the mobilities of the binary complexes approximate that of the labeled protein (Rf = 0.09). However, when both aptamers are incubated with Alexa-thrombin the ternary complex band mobility is retarded to an Rf of 0.08. Therefore, in the context of the system corresponding to the solid phase sandwich the solution study indicates that the ternary complexes are formed in all cases investigated and the system can be applied to the solid phase.

### 3.4. Sandwich Aptamer Microarray Assays: Optimization of the Aptamer-Thrombin Sandwich Protocol

The sandwich system with the modified aptamers analyzed in solution was finally applied to the microarray system, *i.e.*, in solid phase. The SAM was performed with the same system shown in Figure 5, *i.e.*, using TBA1(12T)NH_2_ as capture layer for thrombin and the fluorescently labeled TBA2Cy5 as detection layer. In this case sandwich formation is detected by the red fluorescence of TBA2Cy5.

Alexa-labelled thrombin was also used as control to further verify the sandwich formation by the yellow fluorescence resulting from the co-localization of green (Alexa conjugated to the protein) and red (Cy conjugated to the oligonucleotide) fluorophores. Two different protocols were tested for the sandwich formation on surface: a one-step procedure by deposing on the capture layer thrombin(s) pre-incubated with the detection aptamer (chambers 1 and 2) and a two-steps procedure deposing thrombin on the capture layer, followed by a final deposition of the detection layer. The slides carefully rinsed with the PBS Buffer and MilliQ H_2_O were finally spin-dried and scanned. The samples (six spots for each sample) were prepared as shown schematically in Table 3; each sub-array was physically isolated from the others.

**Figure 6.**
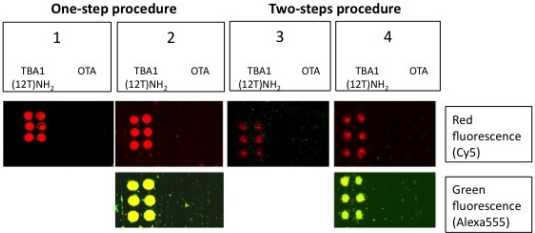
Images of the slide after the Sandwich Aptamer Microarray (SAM). In each sub-array TBA1(12T)NH_2_ was printed on the left, while OTA on the right. The fluorophore Cy5 emitted red light, while Alexa555 emitted green light. For this reason red fluorescence represents TBA2Cy5 and green fluorescence represents labeled thrombin. In chambers 2 and 4, in which labeled thrombin was incubated, the fluorescence was detected with two different contrasts to reveal first red light and then green light: yellow spots indicate the simultaneously localization of the two fluorophores.

The results of the SAM experiments are shown in Figure 6.

In chambers 1 and 2 the red spots (TBA2Cy5) in correspondence of the selection aptamer TBA1(12T)NH_2_ anchored on the glass slide reveal that the sandwich was formed with high specificity for the thrombin aptamer: the detection of the Cy5 labeled aptamer is reproducible across the six spots of each sub-array. The recognition is also specific: no signal is detected for the negative control OTA. SAM is formed with thrombin and also with Alexa-labeled thrombin, consistently with the solution studies: in the sub-arrays 2, Alexa-labeled thrombin allows a further control for thrombin presence in the sandwich by the yellow fluorescence resulting from the simultaneous presence of green fluorescence (Alexa555-thrombin) and red fluorescence (TBA2Cy5). The absence of unexpected hybridization between TBA1 and TBA2Cy5 and the formation of a specific complex between Alexa-thrombin and immobilized TBA1 were also verified independently (not shown).

The one-step protocol is more efficient than the two step procedure: the detection of the sandwich is sharp and specific in chambers 1 and 2 compared with chambers 3 and 4. In chamber 3 the signal obtained is weak, while chamber 4 (Alexa-labeled protein) exhibits a higher background and lower selectivity at this ionic strength. As indicated by the solution studies, the recognition of TBACy5 with human thrombin seems to be the critical step in the development of the best protocol for the solid phase system. The one-step protocols ensures enough time to the modified detection aptamer for the formation of the correct binary complex able to expose the protein epitopes recognized by the immobilized capture aptamer. The presence of the fluorescent dye conjugated to the protein has also a negative effect, possibly influencing the pattern mode of recognition aptamer-thrombin, reflected in the loss of specificity of binding. The microarray system protocol of chamber 1 is a successful application of the aptamer sandwich devised, and further optimization of the solid phase microarray system is in process.

## 4. Conclusions

Several sandwich systems for thrombin detection employing aptamers have been recently reported: among them, a very sensitive device described the use of gold nanoparticles detected by ICP-mass spectrometry [15]. Electrochemical detection was employed with gold-immobilized aptamers in a sandwich system using a secondary aptamer labeled with quantum dots [24], or by using a split aptamer approach [25]. Sensitive systems allowing to re-use the aptasensor were developed based on fluorescence detection, either relying on quantum dots [14] or on europium-phenanthroline chelates [13].

The present study reports the development of a Sandwich Aptamer Microarray (SAM) for human thrombin detection using DNA aptamers modified at the 5′-end for the application to the surface of glass slides and for fluorescence detection. An extensive analysis, optimization and validation of binary and ternary complexes formation between the modified aptamers and thrombin in solution was followed by the verification of the aptamer sandwich formation in solid phase. Our results add information of the effects of modifying aptamers for microarray applications: the 5′-modifications to TBA1 have a minimal but observable effect on thrombin recognition in solution. In solid phase the aptamer orientation through the polyT spacer allows for the correct presentation to the protein, in agreement with the results obtained by other authors analyzing in solid phase the binding of 5′-end modified aptamers to Cy-labeled thrombin [16]. Differently from the co-printing system adopted by these authors, we resorted to a classical sandwich for detecting thrombin. The labeling of TBA2 with the fluorophore Cy5 influences the complex formation with thrombin, as cautioned when reporting a mixed antibody-aptamer format [23]. Our results however show that, overall, the binding to the macromolecular target is preserved in solution when TBA2 is conjugated to Cy5, that was therefore adopted for fluorescent detection. Complex formation was verified analyzing the glass slides by a microarray laser scanner, and correct sandwich formation was confirmed by the fluorescence signals of the sandwich (Figure 6). The microarray for thrombin detection indeed results specific and efficient, therefore representing a potential tool for diagnostic application. The SAM system described here will be further characterized for the limit of detection and optimized for the best incubation time, temperature and presence of protein contaminants by biological samples, to be developed as a simple and sensitive thrombin diagnostic tool with comparable sensitivity and specificity exhibited by known systems [13–16,24,25].

## Figures and Tables

**Table 1.**
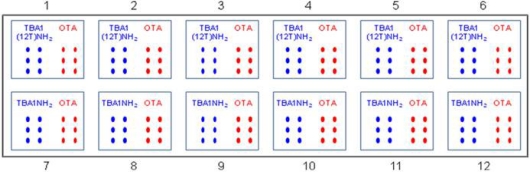
Scheme of the printed glass slide.

**Table 2.** Relative mobilities (Rf) of binary and ternary complexes.

	**+TBA1(12T)NH_2_**		**+TBA2Cy5**		**+TBA1(12T)NH_2_+TBA2Cy5**			
**Protein**	Thr	Alexa555-Thr	Thr	Alexa555-Thr	Thr	Alexa555-Thr	Thr	Alexa555-Thr
**Rf**	0.05	0.09	0.05	0.09	0.04	0.08	0.07	0.09

**Table 3.** Schematic representation of the samples incubated in the SAM protocol.

**Sub-array**	**1**		**2**		**3**		**4**	
**Capture layer**	TBA1(12T)NH_2_	OTA	TBA1(12T)NH_2_	OTA	TBA1(12T)NH_2_	OTA	TBA1(12T)NH_2_	OTA
**Protein**	Thr(0.5 μM) pre-complexed with TBA2Cy5 (1 μM)		Alexa555-Thr (0.5 μM) pre-complexed with TBA2Cy5 (1 μM)		Thr (0.5 μM)		Alexa555-Thr (0.5 μM)	
**Detection layer**					TBA2Cy5 (1 μM)		TBA2Cy5 (1 μM)	
**Procedure**	One-step				Two-steps			

## Abbreviations

Alexa: Alexa555^®^ fluorophore
Cy5: Cyanine dye
DMSO: Dimethyl sulfoxide
EMSA: Electrophoretic Mobility Shift Assay
FBD: Fibrinogen Binding Domain
G-4: G-quadruplex
HBD: Heparin Binding Domain
NAS: *N*,*N*-acryloyloxysuccinimide
OTA: Ochratoxin A binding aptamer
PAA: polyacrylamide
PBS: Phosphate Buffered Saline
SAM: Sandwich Aptamer Microarray
SELEX: Systematic Evolution of Ligands by EXponential enrichment
SDS: Sodium Dodecyl Sulfate
TBA1: unmodified 15-mer Thrombin Binding Aptamer 1
TBA1-NH_2_: 5′-amino modified Thrombin Binding Aptamer 1
TBA1(12T)NH_2_: 5′-amino modified plus a polyT(12) spacer Thrombin Binding Aptamer 1
TBA2: unmodified 29-mer Thrombin Binding Aptamer 2
TBA2Cy5: 5′-Cy5 modified Thrombin Binding Aptamer 2
TBE: tris-borate-EDTA buffer
Thr: Thrombin
